# Diversification of Ergot Alkaloids in Natural and Modified Fungi

**DOI:** 10.3390/toxins7010201

**Published:** 2015-01-20

**Authors:** Sarah L. Robinson, Daniel G. Panaccione

**Affiliations:** Division of Plant and Soil Sciences, West Virginia University, Morgantown, WV 26506, USA; E-Mail: srobins9@mix.wvu.edu

**Keywords:** ergot alkaloids, *Claviceps*, *Epichloë*, *Neosartorya fumigata*, old yellow enzyme, peptide synthetase, prenyl transferase

## Abstract

Several fungi in two different families––the Clavicipitaceae and the Trichocomaceae––produce different profiles of ergot alkaloids, many of which are important in agriculture and medicine. All ergot alkaloid producers share early steps before their pathways diverge to produce different end products. EasA, an oxidoreductase of the old yellow enzyme class, has alternate activities in different fungi resulting in branching of the pathway. Enzymes beyond the branch point differ among lineages. In the Clavicipitaceae, diversity is generated by the presence or absence and activities of lysergyl peptide synthetases, which interact to make lysergic acid amides and ergopeptines. The range of ergopeptines in a fungus may be controlled by the presence of multiple peptide synthetases as well as by the specificity of individual peptide synthetase domains. In the Trichocomaceae, diversity is generated by the presence or absence of the prenyl transferase encoded by *easL* (also called *fgaPT1*). Moreover, relaxed specificity of EasL appears to contribute to ergot alkaloid diversification. The profile of ergot alkaloids observed within a fungus also is affected by a delayed flux of intermediates through the pathway, which results in an accumulation of intermediates or early pathway byproducts to concentrations comparable to that of the pathway end product.

## 1. Introduction

Ergot alkaloids are secondary metabolites synthesized by members of the Clavicipitaceae and Trichocomaceae and have been both harmful and beneficial to humans for thousands of years [1]. Since ancient times, midwives would give the sclerotia of *Claviceps purpurea* to pregnant women to induce labor and reduce bleeding. Currently lysergic acid-derived drugs are used to treat maladies such as Alzheimer’s disease, dementia, type 2 diabetes, and hyperprolactinemia [2,3,4,5]. However, history also has recorded numerous instances of mass ergot poisoning as a result of consumption of ergot-contaminated grain crops; one such instance may have led to the hysteria of the Salem Witch Trials [1]. Similar to humans, wild and domesticated animals have become ill and/or died after ingestion of forage grasses or grain products harboring ergot alkaloids. Grazing animals that consume grasses containing either parasitic or endophytic clavicipitaceous fungi exhibit reduced fitness and weight gain [6]. In the United States alone, it is estimated that hundreds of millions of dollars of losses to the livestock industry are incurred annually by the accumulation of ergot alkaloids in forages [7].

**Figure 1:**
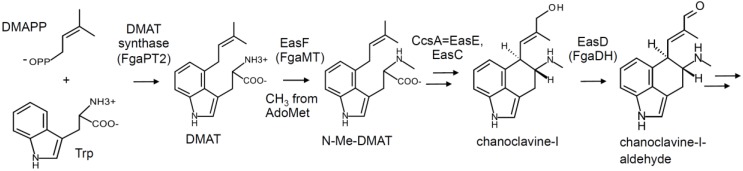
Early steps of the ergot alkaloid pathway. AdoMet, *S*-adenosylmethionine; DMAPP, dimethylallylpyrophosphate.

Members of this diverse class of alkaloids share an ergoline nucleus, which is modified in lineage-specific alkaloid profiles. Despite the Clavicipitaceae and Trichocomaceae being distantly related families, early enzymatic steps of ergot alkaloid biosynthesis are conserved (Figure 1); after the pathways diverge, lineage-specific steps give rise to different profiles of ergot alkaloids in fungi from these two families. Ergot alkaloid producers within the Clavicipitaceae typically produce either lysergic acid-derived ergot alkaloids or dihydroergot alkaloids; several members of the Trichocomaceae produce alkaloids derived from festuclavine. Moreover, within an individual species of fungus, individual isolates can vary in the amount of intermediates produced, total production, and pathway end products. The focus of this review is to describe points of diversification within the pathway leading to distinct profiles of ergot alkaloids among and within species of fungi.

## 2. Pathway Overview and Branch Points

### 2.1. Early, Shared Pathway Steps

All known ergot alkaloid-producing fungi share early steps of the pathway. Roles for the products of *dmaW* in prenylating L-tryptophan (Trp) to dimethylallyltryptophan (DMAT) [8,9,10,11] and *easF* (*fgaMT*) in methylating DMAT to *N*-Me-DMAT [12] have been demonstrated by gene knockout or by heterologous expression in *Escherichia coli* (Figure 1). The necessity of *easC* and *easE* (also called *ccsA*) for converting *N*-Me-DMAT to chanoclavine-I has been demonstrated by gene knockout [13,14], but the exact reactions catalyzed by the products of these genes have not been determined. This set of four genes (*dmaW*, *easF*, *easC*, and *easE*) has been shown to be sufficient to encode the production of chanoclavine-I when expressed in *Emericella nidulans* [15] or *Saccharomyces cerevisiae* [16]. Chanoclavine-I is the first intermediate observed accumulating in many ergot alkaloid producers, and in some cases may accumulate to relatively high concentrations. The product of *easD* (FgaDH) oxidizes chanoclavine-I into chanoclavine-I aldehyde [17], the last shared intermediate and the substrate for the first branch point enzyme (Figure 1).

### 2.2. Branching Caused by Differences in EasA

**Figure 2:**
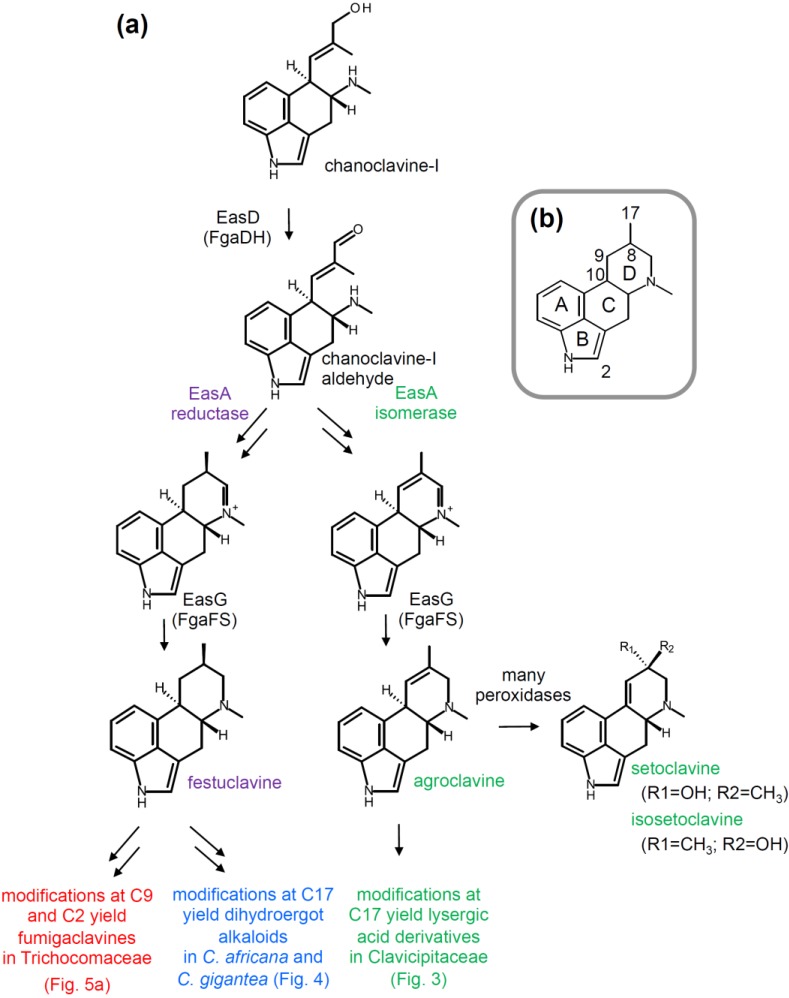
(**a**) Significant branch points in the ergot alkaloid pathway of several fungi. Steps and intermediates common to all ergot alkaloid producers are labeled with black text. The major branch to lysergic acid derived-ergot alkaloids followed by most fungi in the Clavicipitaceae is labeled in green. The branch leading to festuclavine is labeled with purple, and then branches after festuclavine are labeled in blue (for the dihydroergot alkaloids of *C. africana* and *C. gigantea*) or red (for the fumigaclavines of the Trichocomaceae). The same color scheme is used in all figures; (**b**) Position numbering and ring labeling used in text and figures.

After the five conserved pathway steps, chanoclavine-I aldehyde represents the chemical foundation for branching of the ergot alkaloid pathway to produce either festuclavine or agroclavine. Ergot alkaloid-producing fungi in the Trichocomaceae typically produce festuclavine as a key intermediate to more complex ergot alkaloids, whereas most ergot alkaloid-producers in the Clavicipitaceae use agroclavine as a substrate for future modifications. Chemically, the difference between festuclavine and agroclavine is the presence of the 8,9-double bond in agroclavine (Figure 2). To form either of these ergot alkaloids, the aldehyde group of chanoclavine-I aldehyde must be allowed to rotate to come into close proximity with the secondary amine. This rotation is facilitated through the activity of the products of different alleles of *easA* (discussed below), then ring closure to the iminium ion intermediate via Schiff base formation may occur spontaneously. Finally, EasG (also called FgaFS) reduces the iminium ion to produce either festuclavine (with some pyroclavine, as described in Section 4.2) in the Trichocomaceae or agroclavine in typical members of the Clavicipitaceae [18,19,20,21,22,23].

The point of divergence between Clavicipitaceae and Trichocomaceae is dependent on the activities of different versions of the old yellow enzyme called EasA. The form of EasA found in *Neosartorya fumigata* (frequently referred to as *Aspergillus fumigatus* in the ergot alkaloid literature) has activity typical of old yellow enzymes—reduction of a double bond conjugated with either an aldehyde or ketone [18,19,20,21]. On the other hand, the versions of EasA found in most ergot alkaloid producers in the Clavicipitaceae do not permanently reduce the double bond; instead, these enzymes promote isomerization around the double bond [19,20]. We hypothesize that the double bond is temporarily and partially reduced via a mechanism discussed below.

Based on research with purified EasA of *N. fumigata* (EasA_reductase_) and on a wealth of basic studies of other old yellow enzymes [24,25], we propose that the product of the *easA* allele found in *N. fumigata* reduces the 8,9-double bond in chanoclavine-I aldehyde in two steps. First, the flavin mononucleotide cofactor in EasA donates a hydride ion to carbon 9 of chanoclavine-I aldehyde. Next, the reduction is completed when the tyrosine residue at the active site of EasA donates a proton to carbon 8. Versions of EasA found in most Clavicipitaceae lack the tyrosine residue at this position, instead having a phenylalanine [19,20]. As a result of this substitution, the EasA version found in most clavicipitaceous fungi (henceforth EasA_isomerase_) cannot complete the reduction. We hypothesize that the donation of the hydride to carbon 9 allows fleeting rotation resulting in isomerization with retention of the 8,9-double bond. Interestingly, products of the *easA* alleles of *C. gigantea* and *C. africana*––fungi that produce dihydroergot alkaloids via festuclavine––contain the tyrosine residue at the active site [20]. Genetic support of this hypothesis comes from site specific mutations changing the phenylalanine codon to a tyrosine codon in the *E. festucae* var. *lolii* allele of *easA*. This substitution resulted in an enzyme that reduced chanoclavine-I aldehyde as opposed to isomerizing it [20].

Of note is that in the absence of enzymatic activity of EasA, chanoclavine-I aldehyde can be isomerized by nonenzymatic mechanisms including, presumably, keto-enol tautomerization [19] and a reaction involving reduction by glutathione [22]. After isomerization, agroclavine may result from formation of the iminium ion and subsequent reduction by EasG (FgaFS), as described above. In spite of the potential non-enzyamtic isomerization to form agroclavine, EasA_isomerase_ activity contributes significantly to agroclavine formation in genetically modified *N. fumigata*. Evidence for this is the significant increase in agroclavine observed in *easA*_reductase_ knockout (ko) *N. fumigata* strains complemented with *easA*_isomerase_ from *C. purpurea* [19] or *Epichloë festucae* × *typhina* isolate Lp1 [26].

### 2.3. Branch Point after Festuclavine in Dihydroergot Alkaloid Producers

A second branch point separates representatives of the two lineages of fungi that make ergot alkaloids derived from the primary festuclavine branch. These alkaloids are referred to as dihydroergot alkaloids because they have a saturated D ring, whereas the ergot alkaloids of more commonly studied members of the Clavicipitaceae are derived from agroclavine and are unsaturated between carbons 8 and 9 or carbons 9 and 10 (Figure 2). Fungi in the Trichocomaceae, including *N. fumigata* and *Penicillium commune*, produce festuclavine and certain derivatives of festuclavine called fumigaclavines in which carbon 9 is oxidized and/or carbon 2 is prenylated. Note that in the fumigaclavines, carbon 17 remains reduced as a methyl group. Two exceptional members of the Clavicipitaceae—*C. africana*, which causes sorghum ergot, and *C. gigantea*, which causes ergot of maize—produce dihydroergot alkaloids based on oxidation of carbon 17 (in profiles parallel to those observed for more typical members of the Clavicipitaceae that produce ergot alkaloids based on agroclavine). The genetic basis for the branching at festuclavine has not been published, but *C. africana* and *C. gigantea* presumably have alleles of *cloA* that encode enzymes capable of oxidizing carbon 17 of festuclavine, whereas the ergot alkaloid-producing members of the Trichocomaceae lack orthologues of *cloA* [10,11,27]. Members of the Trichocomaceae require a different oxidase to oxidize carbon 9 to produce fumigaclavine B, an acetyl transferase (FgaAT) to acetylate fumigaclavine B to fumigaclavine A [28], and prenyl transferase EasL (FgaPT1) for prenylating carbon 2 of fumigaclavine A (and other ergot alkaloids) [29].

## 3. Diversification within the Clavicipitaceae

### 3.1. Diversity Generated by Peptide Synthetases

**Figure 3:**
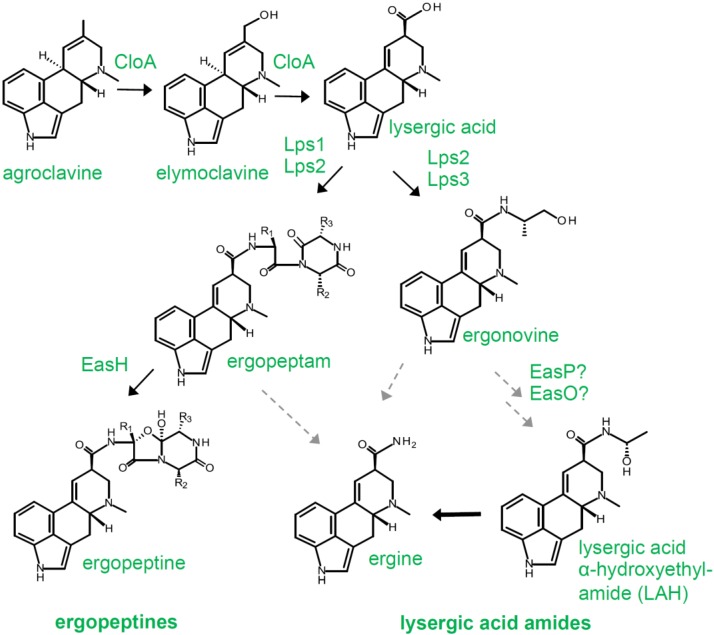
Diversification in the major branch of the ergot alkaloid pathway occurring in the Clavicipitaceae. Steps represented by arrows without enzyme labels occur spontaneously. Dashed arrows indicate hypothesized steps that have not been experimentally demonstrated. R groups in the ergopeptine structure represent side chains for amino acids listed in Table 1.

Most ergot alkaloid-producing members of the Clavicipitaceae have profiles dominated by ergopeptines and/or lysergic acid amides. The diversity among these lysergic acid derivatives arises via an interesting combinatorial system involving two different pairs of peptide synthetases. The peptide synthetase Lps2 (encoded by *lpsB*) [30] has a central role in both enzyme complexes [31,32]. Lps2 recognizes lysergic acid as a substrate and activates it by adenylation for incorporation into either ergopeptines (through interaction with Lps1, encoded by *lpsA*) [33,34] or lysergic acid amides (through interaction with Lps3, encoded by *lpsC*) (Figure 3) [32].

Ergopeptines contain three amino acids linked to lysergic acid (Figure 3). Different ergopeptines vary by the amino acid that occupies each of the three amino acid positions (Table 1). The presence of a particular amino acid at a given position is largely due to the enzyme specificity of individual adenylation domains that recognize and activate the amino acids for incorporation into the ergopeptine. However, there is likely some overlap in amino acid recognition by certain adenylation domains allowing, for example, L-Ile substitution for L-Leu during certain rounds of synthesis. This sort of relaxed specificity has been documented for other peptide synthetases [35], and the presence of small quantities of a variety of ergopeptines in extracts of individual ergot sclerotia is consistent with amino acid substitutions occurring during ergopeptine biosynthesis. The diversity of ergopeptines observed in *C. purpurea* is greater than that observed in *Epichloë* species. The ergot alkaloid synthesis (*eas*) cluster of *C. purpurea* possesses two different copies of *lpsA* [36,37,38], allowing an individual isolate to make two primary ergopeptines (e.g., ergotamine and ergocristine) and then potential variants of each with occasional amino acid substitutions due to relaxed specificity of individual adenylation domains in a given gene.

**Table 1:** Amino acid composition of some common ergopeptines.

Ergopeptine	Amino acid 1	Amino acid 2	Amino acid 3
Ergocristine	l-Val	l-Phe	l-Pro
Ergocryptine	l-Val	l-Leu	l-Pro
Ergocornine	l-Val	l-Val	l-Pro
Ergotamine	l-Ala	l-Phe	l-Pro
Ergovaline	l-Ala	l-Val	l-Pro
Ergosine	l-Ala	l-Leu	l-Pro
Ergobalansine	l-Ala	l-Leu	l-Ala

In addition to ergopeptines, certain members of the Clavicipitaceae produce simple amides of lysergic acid, including ergonovine, lysergic acid α-hydroxyethylamide (LAH), and ergine (Figure 3). Ortel and Keller [32] demonstrated that ergonovine arises via the interaction of Lps2 with Lps3, a monomodular peptide synthetase that recognizes and activates L-Ala. Lps3 has a reductase domain near its carboxy terminus that reduces the carbonyl group to its primary alcohol form, releasing ergonovine from the enzyme. The steps in the biosynthesis of LAH have not been determined, but comparative genomics indicates that it probably arises via the activities of Lps2 and Lps3 and may involve the activities of the products of two additional genes, *easO* and *easP*, whose presence correlates with the ability to produce LAH [38]. Ergine can arise by spontaneous hydrolysis of LAH [39], ergonovine, and ergopeptines, or perhaps their ergopeptam precursors [40,41].

In *Epichloë* spp., homologs of *lpsA* [34] and *lpsB* [42] encode enzymes that function similarly to those of *C. purpurea*; however, ergovaline is the only ergopeptine observed, indicating that a single allele of *lpsA* in *Epichloë* species encodes an enzyme with high fidelity in substrate recognition by its adenylation domains. Homologs of *lpsC*, *easO*, and *easP* are found in *E. gansuensis* var. *inebrians* [43], which produces ergonovine and LAH, though these particular alleles have not yet been functionally analyzed in this species.

Plants in the morning glory family (Convolvulaceae) have symbotic relationships with fungi in the genus *Periglandula*, best exemplified by *Periglandula ipomoeae* in *Ipomoea asarifolia* and *P. turbinae* in *Turbina corymbosa* [44]. The *Periglandula* spp. that have been studied produce diverse profiles of ergot alkaloids often containing lysergic acid amides, such as ergonovine, LAH, and ergine [45,46,47], and the ergopeptine ergobalansine [47]. The genome of *P. ipomoeae* contains a cluster of ergot alkaloid biosynthesis genes, including peptide synthetase genes *lpsA*, *lpsB*, and *lpsC*, that is very similar to those found in the genomes of *C. paspali* and *C. purpurea* [38].

### 3.2. Clavine vs. Lysergic Acid-Based Pathways

Among the members of the Clavicipitaceae that produce ergot alkaloids derived from agroclavine, most of the between-species diversity is generated by the incorporation of lysergic acid into different ergopeptines or different lysergic acid amides (as described in Section 3.1); however, there are examples of clavicipitaceous fungi that generate unique profiles by terminating their pathways at atypical points. One example is that some isolates of *Epichloë elymi* and *Epichloë canadensis* terminate their pathways at chanoclavine-I, and genome sequencing demonstrates that these isolates have only *dmaW*, *easF*, *easC*, and *easE* in their genomes [38,43]. Another example is *Claviceps fusiformis*, which accumulates agroclavine and elymoclavine [48,49]. The ergot alkaloid synthesis gene cluster of this fungus contains a copy of *lpsB* that is clearly a pseudogene, and the genome lacks homologs of *lpsA* and *lpsC*. The copy of *cloA* in *C. fusiformis* is transcribed and has sequence characteristics that would appear to allow it to encode a functioning protein; nonetheless, the gene product appears to be incapable of catalyzing lysergic acid formation, since it is unable to complement the *cloA* knockout of *C. purpurea* [49].

Whereas some isolates of *Claviceps paspali* have the capacity to produce lysergic acid amides, *C. paspali* NRRL 3080, isolated from *Paspalum dilatatum* in Portugal, accumulates primarily paspalic acid (the Δ8,9 isomer of lysergic acid) with smaller quantities of lysergic acid and isolysergic acid, which have been presumed to arise by spontaneous isomerization of paspalic acid [50,51]. Indeed, paspalic acid can spontaneously isomerize to lysergic acid over time, and the process can be accelerated by base and/or heat [50]. However, the existence of a paspalic acid accumulating variant of *C. paspali* [50] and the lack of detectable paspalic acid in strains of *N. fumigata* engineered to accumulate lysergic acid [26] suggest that in a properly functioning ergot alkaloid pathway, the isomerization is enzymatically catalyzed. The genetic basis for the truncation of the pathway of *C. paspali* NRRL 3080 at paspalic acid is presently unknown.

### 3.3. Dihydroergot Alkaloids in Certain Members of the Clavicipitaceae

**Figure 4:**
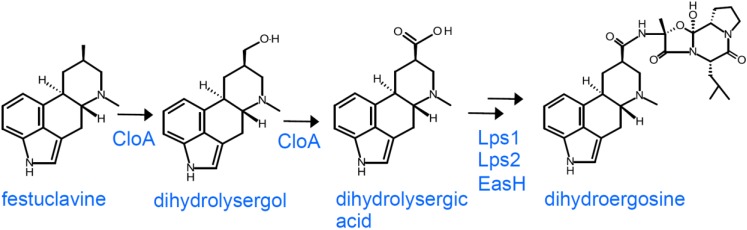
Pathway of dihydroergot alkaloids in *C. africana* or *C. gigantea*. The pathway in *C. gigantea* ends at dihydrolysergol, whereas that of *C. africana* extends to dihydroergosine. Roles for all enzymes are hypothesized based on analogy with *Epichloë* species and *C. purpurea*.

The two members of the Clavicipitaceae (*C. africana* and *C. gigantea*) that produce dihydroergot alkaloids via the festuclavine branch of the pathway follow a linear pathway without generating much diversity (Figure 4). *Claviceps gigantea* terminates its pathway at dihydrolysergol, the first oxidation product of festuclavine [52], whereas *C. africana* completes the oxidation of festuclavine to dihydrolysergic acid and incorporates dihydrolysergic acid exclusively into the dihydroergopeptine dihydroergosine [53,54].

## 4. Diversification within the Trichocomaceae

### 4.1. Diversification Based on Presence and Relaxed Specificity of Prenyl Transferase EasL

In the Trichocomaceae there are fewer characterized producers of ergot alkaloids than in the Clavicipitaceae, which inevitably reduces diversity currently documented within this family. Several species in Trichocomaceae make ergot alkaloids including the human pathogenic fungus *N. fumigata*, common dairy contaminant *P. commune*, and soil fungus *Aspergillus japonicus* [27,41,55]; however, relatively few of these have been studied from a molecular perspective. Unless otherwise specified, alkaloid data reviewed here will be from *N. fumigata*.

**Figure 5:**
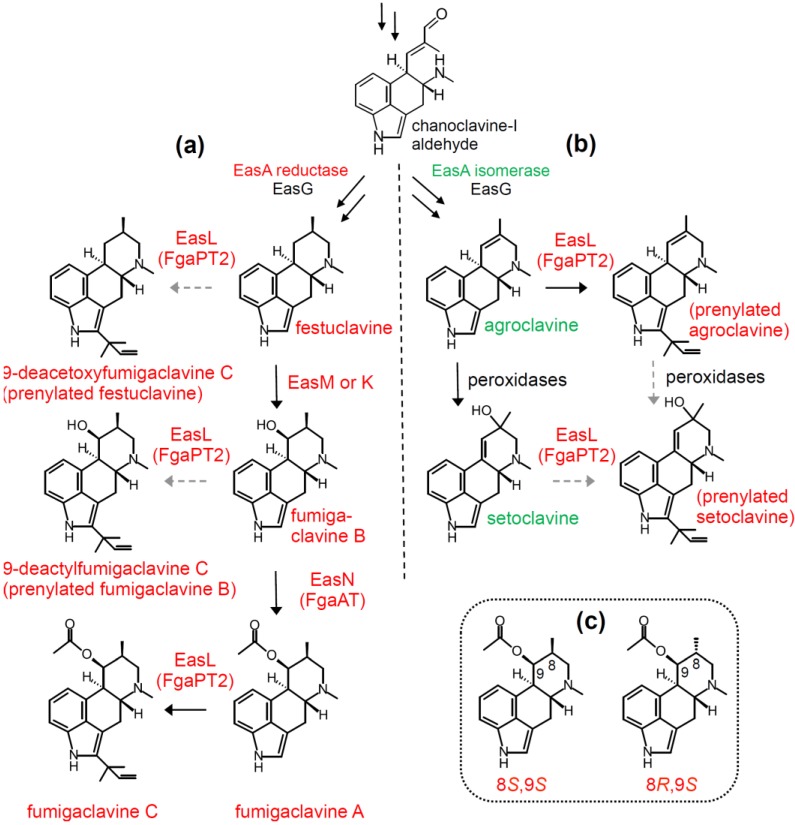
(**a**) Diversification of ergot alkaloids in the Trichocomaceae. Some isolates of *N. fumigata* terminate their pathway at fumigaclavine A, whereas others produce fumigaclavine C [56]. *Penicillium commune* lacks *easL* [27] and terminates its pathway at fumigaclavine A. The role of prenyl transferase EasL (also called FgaPT1) in producing fumigaclavine C (and presumably 9-deacetoxyfumigclavine C and 9-deactylfumigaclavine C) is indicated; (**b**) Pathway resulting from expression of *easA*_isomerase_ in *N. fumigata* *easA* ko. The role of EasL in prenylating agroclavine [26] is indicated. Peroxidases common to *N. fumigata* and many organisms [40,60] oxidize agroclavine to setoclavine. Alternate routes for production of prenylated setoclavine [26] are indicated. (**c**) 8*S*,9*S* fumigaclavine A compared to 8*R*,9*S* fumigaclavine A. Alkaloids typically found in the Clavicipitaceae are labeled with green. Dashed arrows indicate hypothesized steps.

Starting at the branch point between Trichocomaceae and most Clavicipitaceae, *N. fumigata* typically follows a pathway in which festuclavine is converted ultimately into fumigaclavine C following oxidation of festuclavine to fumigaclavine B, acetylation of fumigaclavine B into fumigaclavine A, and finally reverse prenylation of fumigaclavine A to fumigaclavine C (Figure 5). Most studies of ergot alkaloids in *N. fumigata* have focused on two well-characterized strains: Af 293 and FGSC A1141. Both strains synthesize fumigaclavine C as their final ergot alkaloid. Robinson and Panaccione [56] reported that *N. fumigata* isolates collected from different geographical locations around the world displayed diverse ergot alkaloid profiles. Although the authors found no association between geographical location and chemotype (perhaps due to the small sample size), they did observe that the majority of the diversity fell into two categories: fumigaclavine A producers and fumigaclavine C producers. After sequencing *easL (fgaPT1)* in the fumigaclavine A producers, the authors found that four of seven fumigaclavine A producers had premature stop codons, resulting in a severely truncated protein. However, three fumigaclavine A-accumulating isolates had *easL* sequences with no obvious defects but did not continue through the pathway to fumigaclavine C. The subsequent discovery that peptide synthetase genes *pesL* and *pesI* were necessary for the biosynthesis of fumigaclavine C [57] may provide an explanation for this apparent paradox. Lack of function in either of these peptide synthetases phenocopies EasL (FgaPT1) deficiency. Overall, the data imply that in some environments there may be an advantage of one end product over the other (fumigaclavine A *vs.* fumigaclavine C) or at least that there is no selection against strains that have lost a functional *easL*. The chemotype of *P. commune* is similar to that of *N. fumigata*, except that *P. commune* lacks *easL* [27] and thus terminates its pathway at fumigaclavine A and produces no other 2-prenylated ergot alkaloids. Moreover, the stereochemistry of the fumigaclavines in *P. commune* differs from that in *N. fumigata* [23], as described in Section 4.2.

Further opportunities for diversification of the *N. fumigata* ergot alkaloid profile come from the relaxed specificity of EasL (FgaPT1). Originally characterized as the enzyme required for reverse prenylation of fumigaclavine A into fumigaclavine C [29], recent studies have indicated other compatible substrates. For example, Ge *et al.* [58] described novel ergot alkaloids named 9-deacetoxyfumigaclavine C and 9-deacetylfumigaclavine C (Figure 5). Although the biosynthetic origin of these compounds was not analyzed or speculated upon by Ge *et al.* [58], we propose that 9-deacetoxyfumigaclavine C and 9-deacetylfumigaclavine C are derived from EasL-catalyzed prenylation of festuclavine and fumigaclavine B, respectively. In a recent study, Xu *et al.* [59] reported several additional alkaloids that were prenylated (and in some cases demethylated) versions of stereoisomers of festuclavine, fumigaclavine B, and fumigaclavine A. The hypothesis of prenylation of multiple substrates by EasL (FgaPT1) is supported by recent data involving pathway manipulation in *N. fumigata*. Taking advantage of the *easA* branch point, Robinson and Panaccione [26] reprogrammed *N. fumigata* to produce agroclavine and setoclavine, which are typically produced in the Clavicipitaceae. In addition to agroclavine and setoclavine, the modified *N. fumigata* strains produced two novel alkaloids which, based on spectral analysis, the authors hypothesized to be 2-reverse-prenylated versions of agroclavine and setoclavine. Because EasL reverse prenylates fumigaclavine A to fumigaclavine C, the authors further hypothesized that EasL was acting on agroclavine and setoclavine. Using a natural variant that fails to prenylate fumigaclavine A into fumigaclavine C as a result of a premature stop codon in *easL* [56], the authors found that production of the prenylated forms of agroclavine and setoclavine was dependent on a functional copy of *easL* (Figure 5). Collectively, these studies indicate that agroclavine, setoclavine, festuclavine, fumigaclavine B, and fumigaclavine A can all be substrates for EasL. Alternatively, the prenylated version of setoclavine could arise from oxidation of the prenylated version of agroclavine via the activity of non-specific oxidases that typically act on agroclavine (Figure 5). Of note, in experiments in which lysergic acid was expressed in the same background as the prenylated agroclavine and setoclavine, there was no evidence of prenylated lysergic acid. In addition, strains that accumulate chanoclavine-I and chanoclavine-I aldehyde [19] also do not have evidence of prenylated versions of these intermediates. One common feature among lysergic acid, chanoclavine-I, and chanoclavine-I aldehyde is the presence of oxidized carbon 17, which may impact the suitability of these alkaloids as substrates for EasL. Similar observations were previously noted by Unsöld and Li [29], who documented that EasL failed to modify several lysergic acid-derived alkaloids (which also are oxidized at carbon 17).

### 4.2. Stereochemical Diversification Based on Activity of EasG (FgaFS)

Whereas, the majority of fumigaclavines are in the 8*S*,9*S* configuration, a minority are in the 8*R*,9*S* configuration (Figure 5), probably because they are derived from the C8 stereoisomer of festuclavine, which is called pyroclavine. Versions of EasG (FgaFS) from *N. fumigata* and *P. commune* differ in the proportion of the stereoisomers in their products [23]. *In vitro*, the product of the allele from *N. fumigata* produced festuclavine and pyroclavine at a 95:5 ratio, whereas the product of the allele from *P. commune* produced festuclavine to pyroclavine at a ratio of 79:21. Interestingly, in *P. commune*, only pyroclavine was further metabolized and ultimately was converted into an 8*R*,9*S* isomer of fumigaclavine A (Figure 5), presumably because pyroclavine but not festuclavine served as a substrate for the downstream oxidase in the pathway [23]. Evidence for the existence of 8*R*,9*S* isomers of fumigaclavines in *N. fumigata* (in addition to the more abundant 8*S*,9*S* isomers) was recently provided by Xu *et al.* [59] who detected prenylated versions of pyroclavine and 8*R*,9*S* fumigaclavine B.

### 4.3. Diversification via Multiple Enzymes Competing for a Common Substrate

The production of agroclavine in engineered *N. fumigata*, a fungal species that naturally does not accumulate this compound, provides an interesting opportunity to observe the fate of agroclavine and derivatives of agroclavine resulting from its metabolism by several competing enzymes. In the absence of CloA, which oxidizes agroclavine to lysergic acid [26,51], agroclavine that accumulates in engineered *N. fumigata* may stay in its original form, be oxidized to setoclavine [19,26], or be prenylated [26]. The greater abundance of setoclavine compared to the prenylated forms indicates that activity or abundance of nonspecific oxidases exceed that of the reverse prenyl transferase EasL (FgaPT1). Another possible explanation is that limits on DMAPP availability cause prenylation to proceed slower *in vivo*. When CloA is expressed in this same background, most of the available agroclavine is incorporated into lysergic acid, whereas the residual agroclavine is distributed among products in proportions similar to those observed in the absence of CloA.

## 5. Diversification of Chemotype via Delayed Flux and Spur Products

### 5.1. Inefficiency or Delayed Flux Can Yield Multiple de Facto Products from a Single Pathway

Diversity of ergot alkaloid profiles among different ergot alkaloid producers is generated by the pathway branches described above. Within an individual organism, however, diversity in the profile of ergot alkaloids detected at any given time can be generated by inefficiency, or delayed flux, in converting one pathway intermediate to the next. In some fungi, certain intermediates may accumulate to concentrations on the same order of magnitude as the end product of that pathway. Certain intermediates, if left to accumulate, are oxidized or otherwise metabolized to ‘spur’ products—the terminal products of short branches from the pathway (e.g., setoclavine as an oxidation product of agroclavine in Figure 2). Thus, the *de facto* output of the ergot alkaloid pathway includes high-accumulating intermediates and their spur products in addition to pathway end products.

### 5.2. Delayed Flux in the Clavicipitaceae

Fungi in the Clavicipitaceae differ in their efficiency in converting one intermediate to the next in the ergot alkaloid pathway. Certain *Epichloë* species accumulate relatively high concentrations of intermediates and spur products. For example, *E. festucae* × *typhina* Lp1 in perennial ryegrass accumulates the early pathway intermediate chanoclavine-I and/or early pathway spur products to concentrations that approach [40,61] or sometimes exceed [62,63] the concentration of pathway end product ergovaline. An interesting *post hoc* observation is that in studies in which leaf blades were analyzed [62,63], concentrations of intermediates and spur products exceeded the concentration of ergovaline; whereas, when pseudostems were analyzed [40,61], ergovaline was the most abundant alkaloid. These data must be interpreted with caution, however, because in these four studies, both tissue types were not analyzed from the same individual plants. Symbiotic *Periglandula* species associated with morning glories also exhibit reduced flux in their ergot alkaloid pathways, often accumulating relatively high concentrations of the intermediate chanoclavine-I in addition to multiple end products including simple amides of lysergic acid and the ergopeptine, ergobalansine [45,46,47]. *Claviceps purpurea* appears more efficient in completing its ergot alkaloid pathway, as intermediates are not frequently detected at high concentrations; however, *C. purpurea* generates diversity by incorporating lysergic acid into ergonovine and one or more ergopeptines.

The accumulation of intermediates in ergot alkaloid producers does not appear to be random, as chanoclavine-I often accumulates (particularly in *Epichloë* spp. and in morning glories symbiotic with *Periglandula* spp.), but other intermediates are detected less frequently and less abundantly. Two potential reasons for this apparent selectivity in intermediate accumulation are (1) that chanoclavine-I provides some benefit to the producing fungus or its host plant or (2) that other intermediates cannot be allowed to accumulate due to toxicity to the producing fungus or its host. Comparison of results of studies with gene knockout mutants of the perennial ryegrass endophyte *E. festucae* × *typhina* Lp1 provides some evidence that chanoclavine-I or other early pathway products have activities that differ from those of the pathway end products derived from lysergic acid. Rabbits are deterred from feeding on perennial ryegrass containing mutants of *E. festucae* × *typhina* Lp1 that cannot produce lysergic acid derivatives but do accumulate chanoclavine-I and other early pathway clavines, but mutants lacking all ergot alkaloids lose their ability to deter feeding by rabbits [62]. Conversely, mutants retaining chanoclavine-I but lacking lysergic acid derivatives lose their ability to kill and deter feeding by black cutworm (*Agrotis ipsilon*) [63].

Some hints on the regulation of the ergot alkaloid pathway can be gleaned from studies on the *lpsA* knockout mutant of *E. festucae* × *typhina* Lp1. When *lpsA* is knocked out, the overall molar yield of the ergot alkaloid does not change, but the distribution of ergot alkaloids along the pathway changes such that alkaloids that were distributed among clavine and lysergic acid derivatives in the wild type accumulate as clavine intermediates or spur products in the mutant [40,61,62,63].

### 5.3. Delayed Flux in the Trichocomaceae

In isolates of *N. fumigata* capable of producing fumigaclavine C, several earlier pathway intermediates accumulate to relatively high levels. These include festuclavine, fumigaclavine A, fumigaclavine B, and chanoclavine-I (Figure 5) [56,64]. Similarly, isolates that terminate their pathways at fumigaclavine A accumulate festuclavine, fumigaclavine B, and sometimes chanoclavine-I to detectable levels [56]. Relative efficiency in turning over intermediates to complete the pathway varies from strain to strain of *N. fumigata*; in some cases, the intermediate festuclavine was the most abundant ergot alkaloid measured in some fumigaclavine C producers and some fumigaclavine A producers [56].

## 6. Conclusions

Different classes of ergot alkaloids are produced by fungi from two distinct lineages. Most ergot alkaloid producers in the Clavicipitaceae produce ergot alkaloids derived from lysergic acid, whereas those in the Trichocomaceae produce festuclavine and fumigaclavines which differ from lysergic acid-derived ergot alkaloid in saturation of the D ring and lack of oxidation at carbon 17 (Figure 2). The major branch point between these two lineages is determined by the allele of *easA* in the respective lineages. The ergot alkaloid producers in the Trichocomaceae, as well as *C. africana* and *C. gigantea* (atypical members of the Clavicipitaceae), possess the reductase form of EasA, leading them down a pathway to dihydroergot alkaloids (containing a saturated D ring). The majority of ergot alkaloid producers in the Clavicipitaceae have an isomerase form of EasA, which allows them to follow a pathway to the lysergic acid derived ergot alkaloids.

In the Clavicipitaceae, multiple enzymes acting on lysergic acid and amino acids diversify the chemotypes observed. Conversely, in the Trichocomaceae, one prenyl transferase (EasL = FgaPT1) acting on multiple substrates leads to chemotypic diversification. In both lineages, the delayed flux of ergot alkaloids through the pathway results in an accumulation of particular intermediates that further diversify the observed chemotypes and contribute to the production of multiple products from a single pathway. One implication of this pattern of alkaloid accumulation is that different ergot alkaloids provide different benefits to the fungi that produce them.
